# Statistical Dissection of Cyto-Nuclear Epistasis Subject to Genomic Imprinting in Line Crosses

**DOI:** 10.1371/journal.pone.0091702

**Published:** 2014-03-18

**Authors:** Tao He, Jian Sa, Ping-Shou Zhong, Yuehua Cui

**Affiliations:** 1 Department of Statistics and Probability, Michigan State University, East Lansing, Michigan, United States of America; 2 Division of Medical Statistics, School of Public Health, Shanxi Medical University, Taiyuan, Shanxi, China; Pennsylvania State University, United States of America

## Abstract

Cytoplasm contains important metabolism reaction organelles such as mitochondria and chloroplast (in plant). In particular, mitochondria contains special DNA information which can be passed to offsprings through maternal gametes, and has been confirmed to play a pivotal role in nuclear activities. Experimental evidences have documented the importance of cyto-nuclear interactions in affecting important biological traits. While studies have also pointed out the role of interaction between imprinting nuclear DNA and cytoplasm, no statistical method has been developed to efficiently model such effect and further quantify its effect size. In this work, we developed an efficient statistical model for genome-wide estimating and testing the cytoplasmic effect, nuclear DNA imprinting effect as well as the interaction between them under reciprocal backcross and F_2_ designs derived from inbred lines. Parameters are estimated under maximum likelihood framework implemented with the EM algorithm. Extensive simulations show good performance in a variety of scenarios. The utility of the method is demonstrated by analyzing a published data set in an F_2_ family derived from C3H/HeJBir and C57BL/6 J mouse strains. Important cyto-nuclear interactions were identified. Our approach provides a quantitative framework for identifying and estimating cyto-nuclear interactions subject to genomic imprinting involved in the genetic control of complex traits.

## Introduction

One of the central foci in biological study is to unravel the genetic secrets of complex traits of agricultural, evolutional and biomedical importance. Quantitative trait locus (QTL) mapping has been the major tool for this purpose over decades [1]–[3]. In QTL mapping, the identified QTLs are chromosome segments harboring potential genetic variants that could give rise to phenotypical manifestation. Large successes have been witnessed in the past [4]. However, there are still many phenomena that could not be explained by Mendelian genetics, leading to the new exploration of research focus on epigenetics [5].

Genomic imprinting, one of the major epigenetic phenomena, plays key roles in controlling embryonic growth and development [6], [7]. Let subscript letter 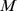 and 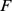 denote the parental origin of two alleles in a diploid organism, then a locus with two alleles 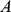 and 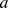 is thought to be imprinted if two heterozygotes 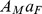 and 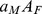 have different expressions [8]. The malfunction of imprinted genes could potentially lead to abnormal characters such as cancers or other genetic disorders [9].

Genomic imprinting effect is considered as one type of parent-of-origin effect due to allelic effect with specific parental origin. In contrast to this, maternal effect or cytoplasmic effect is also considered as one type of parent-of-origin effect in which the offspring's expression is influenced by maternal parent. For example, a mother's genotype, even if not transmitted to her offspring, may influence in utero conditions and increase risk and/or interact with genetic predisposition for particular diseases among those offspring [10]. For cytoplasm, it contains a wide variety of organelles such as mitochondria and chloroplast (in plant). Almost all the reactions of cellular metabolism take place in such an environment. It has been demonstrated that cytoplasm plays a central role in coordinating the activities of nuclear genetic materials [11]–[15]. Thus, the identification of cyto-nuclear interaction could shed novel insights into the genetic and epigenetic control of phenotypic variation. A number of empirical studies have documented the significant contribution of cyto-nuclear interaction to phenotypic variation in organisms such as wheat, rice, mice, yeast and Drosophila [16]–[19].

On the other hand, the existence of such parent-of-origin effects may lead to incorrect interpretation of the (marginal) effects of particular genes when performing genetic mapping studies, unless such effects are appropriately accounted for in the analysis [20]. Statistical methods for dissecting genomic imprinting effect has been extensively studied in literature (e.g., [21]–[26]). Tang et al. [27] developed a model to evaluate cyto-nuclear interaction effect based on experimental crosses. However, how the two types of parent-of-origin effects, one in nuclear level and one in cytoplasmic level, interact to influence phenotypic variation is largely unknown due to the lack of proper statistical models.

In this work, we discuss potential scenarios of parent-of-origin effects, and present a statistical method to dissect the cyto-nuclear interaction effects subject to genomic imprinting. The developed framework is based on experimental crosses which can be realized through two different designs, reciprocal backcross and F_2_ design. When an F_2_ design is considered, sex-specific difference in recombination fractions, which is initially discovered in [28], [29] and later observed in many species, is incorporated into our model to distinguish the genetic differences between two reciprocal heterozygote. Such information can be found in literature, such as the female-to-male recombination ratio of 1.6∶1 for human [30], 1.4∶1 for pig [31], 1.4∶1 for dog [32], 1.25∶1 for mouse [33] on average across the whole genome. A genome-wide scan for the identification of iQTL mapping cyto-nuclear epistasis is performed based on the interval mapping theory, and parameters are estimated based on the framework of maximum likelihood method implemented with the EM algorithm [34]. Extensive simulations are conducted to evaluate the performance of our model under different scenarios, such as different sample sizes, different heritability levels, and different gene effects. The utility of the method is illustrated by applying it to a genome-wide scan of four traits in an F_2_ family derived from two inbred mouse strains.

## Statistical Methods

### Genetic designs

Consider a design initiated with two inbreed lines with two segregating alleles 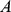 and 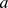. Let subscript letter 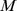 and 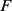 represent the parental origin of offspring alleles inherited from the mother and father, respectively. A complete dissection of the cyto-nuclear interaction subject to imprinting needs experimental designs that can distinguish the quantitative variation between two heterozygotes 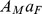 and 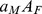, and also against the cytoplasmic effect. For this purpose, a reciprocal backcross or F_2_ design is proposed so that variations and interactions can be fully introduced.

**Figure 1:**
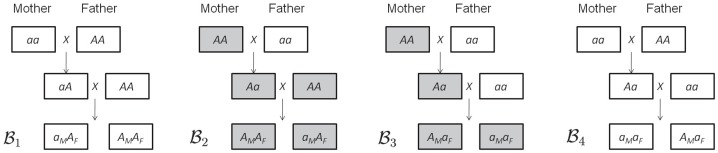
A reciprocal backcross design.

Figure 1 illustrates the reciprocal backcross design. Let the maternal parent carrying genotype 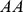 (denoted as P2) has a cytoplasmic effect in contrast to the maternal line carrying genotype 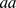 (denoted as P1) as the reference line. In the diagram, individuals that carry the maternal effect coming from the maternal parent with genotype 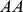 are denoted by gray squares. Four possible backcrosses can be initiated as illustrated in Fig. 1. As shown in the figure, any backcross offsprings coming from the middle two designs carry the cytoplasmic maternal effect derived from the 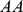 genotype.

**Figure 2:**
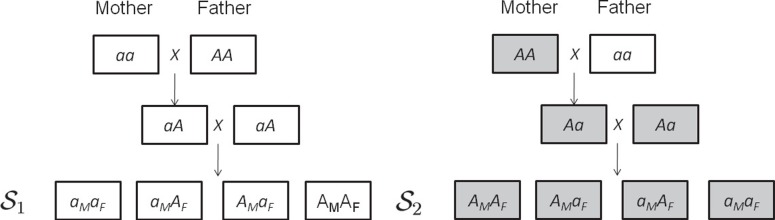
A reciprocal F_2_ design.

Figure 2 shows the F_2_ design. Denote the left one as design 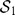, and the right one as design 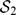. The cross of two F_1_'s generates four possible allele-specific F_2_ genotypes. Assuming there is a cytoplasmic effect, F_2_ offsprings may show different phenotypes depending on the genotype of the maternal parental lines. For example, if maternal cytoplasmic effect exists, the offspring phenotypic value for 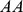 genotype may be different depending on whether it comes from the 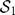 or 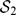 design. In the F_2_ design, the two reciprocal heterozygotes 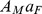 and 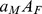 cannot be distinguished in general. Sex-specific recombination difference in male and female needs to be considered in order to distinguish the two (Cui et al. 2006) [25].

### Statistical parameterization

For a particular cross, let 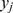 (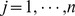) denote the phenotypic value of interest. Following Tang et al. [27], the one-QTL genetic model can be expressed as,
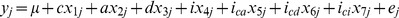
(1)where 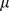 is the overall mean; 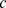 is the cytoplasmic effect; 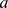, 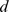 and 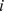 are the additive, dominance and imprinting effects of a QTL, respectively; and 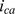, 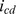 and 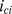 are the cytoplasm by additive, cytoplasm by dominance and cytoplasm by imprinting interactions, respectively; 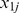 is an indicator variable, denoting 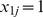 for the 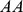 maternal cytoplasm and 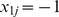 for the 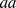 maternal cytoplasm; 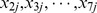 are other indicators of various effects describing the additive, dominance and the interaction effect between the cytoplasm and genetic variables.

For the 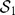 design in the F_2_ population initiated with cross 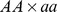, the mean genotypic values of four possible genotypes formed by different allelic combination from the two F_1_ parents can be expressed as,
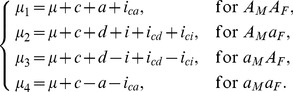
(2)


Similarly for the 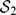 design initiated with cross 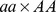, the genetic model can be expressed as,
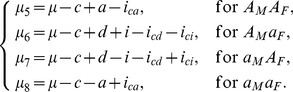
(3)


For the reciprocal backcross design which consists of 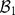, 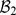, 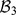 and 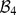, the indicator variables in Eq. (1) describing different QTL genotypes are defined in Table 1.

**Table 1:** QTL genotypes and corresponding genetic components under different backcross designs.

Backcross design	BC QTL genotype	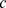	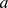	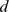	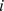	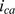	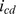	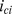
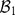	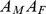	−1	1	0	0	−1	0	0
	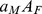	−1	0	1	−1	0	−1	1
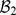	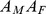	1	1	0	0	1	0	0
	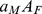	1	0	1	−1	0	1	−1
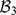	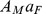	1	0	1	1	0	1	1
	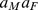	1	−1	0	0	−1	0	0
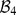	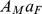	−1	0	1	1	0	−1	−1
	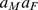	−1	−1	0	0	1	0	0

For simplicity, we will use matrix form to rewrite the models. Let us denote
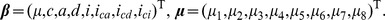
Then the relationship between the eight genetic means and the eight parameters can be written as
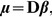
(4)where
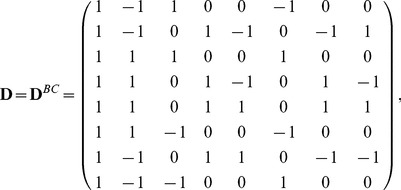
(5)for the reciprocal backcross design, and
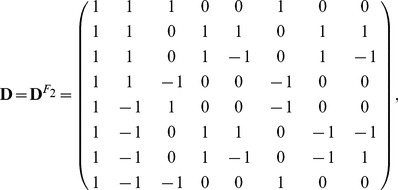
(6)for the F_2_ design. For the purpose of illustration, the following estimation and inference is demonstrated through the F_2_ design. The same procedure applies to BC design too.

### The mixture model and the likelihood

Statistical methods for QTL interval mapping based on a mixture model traced back to the work by Lander and Botstein [1]. In the mixture model, each observation 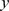 is modeled as a weighted mixture of 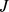 (known and finite) components, and each component, which corresponds to a certain genotype category depending on the underlying genetic design, follows a certain distribution 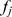 with weight 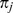. Conditional on the marker genotype **M** and unknown parameters 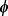 and 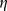, the density of the observed 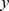 has the following expression
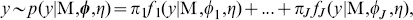
(7)where 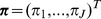 refers to the mixture proportions which are constrained nonnegative and 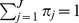; 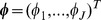 is a vector for the component-specific parameters, with 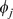 being specific to 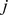
th component; and 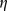 consists of parameters (i.e., residual variance) that are common to all components.

For the F_2_ design we described above, there are four genotypes at each locus (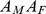, 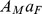, 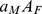, and 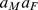). The genotype of the QTL is generally unobservable, but can be inferred by using the two flanking markers' information. Given the flanking marker genotypes of the 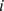
th individual, the conditional probabilities 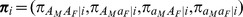 of the QTL genotype can be calculated. These conditional probabilities become the mixture proportions in the mixture model (7). Let us denote 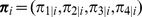 to simplify the notation. These conditional probabilities are expressed in terms of sex-specific recombination rates in order to distinguish the two reciprocal heterozygotes. Please refer to Cui et al. [25] for the conditional probabilities of QTL genotypes given marker genotypes in terms of sex-specific recombination fractions for an F_2_ design.

Assume the total number of F_2_ offsprings for design 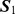 and 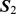 are 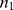 and 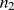 respectively, and let 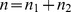. Phenotype data for a certain quantitative trait can be observed and recorded as a vector 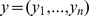, where 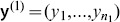 are from design 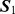 and 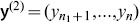 are from design 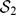. Marker information can be reorganized as matrix 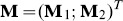, where the 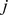
th 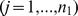 row of 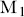 (the 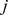
th row of matrix 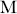) contains all the marker information of the 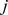
th individual under design 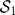 and the 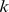
th 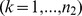 row of 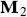 (the 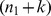
th row of matrix 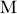) contains all the marker information of the 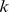
th individual under design 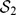. Based on the mixture model (7), and with the independence assumption, the joint likelihood function for the F_2_ family with total 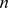 individuals, constructed by combining design 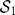 and 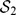 together, can be formulated as,
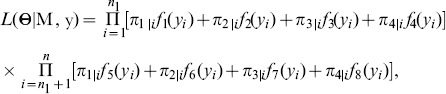
(8)where the unknown vector 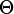 contains the QTL position, QTL effects and residual variance, and the density function 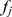 (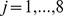) is assumed to follow a normal distribution with mean 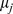 and common variance 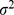. More specifically, parameter vector 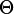 can be divided into two subsets, 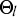 and 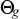, where 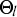 describes the location of QTL and 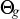 contains all the genetic parameters, including QTL-effects vector 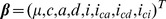 and residual variance 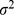 in our model.

### Parameter Estimation

To estimate the unknown parameters 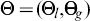, several algorithms could be implemented, such as Expectation-Maximization (EM), Newton Raphson and Fisher Scoring. Among all these methods, EM algorithm, which was initially developed by Dempster et al. [34], is most commonly used in QTL mapping study. In this paper, EM algorithm is applied to obtain the maximum likelihood estimates (MLEs). This procedure involves differentiating the log-likelihood function with respect to each unknown parameter, letting the derivatives equal to zero, and solving the log-likelihood equation for the corresponding parameter. Please read Appendix S1 for the detailed derivations of parameters estimation and the algorithm.

For the QTL position which is unknown in the model, we did not estimate parameters 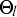 directly. As commonly treated in QTL mapping studies, we applied a grid search approach to estimate the putative QTL position via scanning the entire linkage genome by 1 or 2 cM increment flanked by two markers and did a hypothesis testing at each putative position. A likelihood ratio or LOD profile plot can be generated to graphically display the LR or LOD test statistic for a putative QTL at each testing position. The genomic position which corresponds to a peak in the profile plot is the MLE of the QTL location, given that the peak passes the genome-wide threshold determined by the permutation tests detailed below. Bootstrap methods can be applied to assess the confidence interval of the estimated position [35].

### Hypothesis Test

Testing the overall QTL effect on the quantitative trait is the first step toward a complete dissection of different genetic contributions to the trait. Once the MLEs of the parameters are obtained, the presence of QTL responsible for the variation of the quantitative phenotype can be tested by using the following hypotheses,
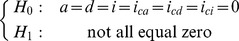
The test statistic for testing the above hypotheses is calculated as the log-likelihood ratio test statistic (LR) of the full model (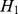) over the reduced model (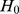):
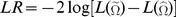
(9)where 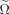 and 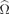 denote the MLEs of the unknown parameters under 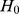 and 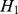, respectively. Since a genome-wide scan involves multiple correlated tests, we use the permutation tests proposed by Churchill and Doerge [36] to find the threshold value.

If there is a QTL, a number of other hypothesis tests can also be performed to test the property of the detected QTL. To test the imprinting effect, we can simply formulate the hypothesis as 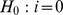 vs 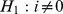 to assess the mean difference of the two reciprocal heterozygotes. To test the cytoplasmic maternal effect, the hypothesis can be stated as 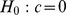 vs 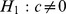. The epistatic effects of all interaction terms can also be tested as
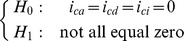
Similarly, additive and dominance effects can be tested as
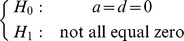
If specific interest is focused, for instance, the interaction of imprinting and maternal effect, the hypothesis can be formulated as 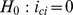 vs 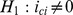. All the above tests can be done by applying the likelihood ratio test in which the test statistic asymptotically follows a 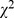 distribution with degrees of freedom equal to the difference of the parameters under the null and the alternative hypotheses. For example, when testing 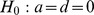, the LR test statistics is compared with the 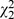 cutoff with 2 degrees of freedom.

## Results

### Monte Carlo simulation

Monte Carlo simulations were performed to investigate the statistical behavior of our model. We simulated an F_2_ population, with one half of the population coming from design 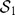 and the other half coming from design 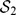. A genome with 100 cM long linkage group, composed of 6 equidistant markers, was constructed. The position of QTL was assumed to be located at 48 cM away from the first marker on the linkage group. The marker genotypes in the F_2_ population were simulated by mimicking sex-specific recombination fractions in mice, i.e., 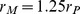 [33]. A series of simulation study with different sample size (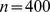 vs 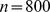) and different heritability levels (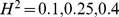) was conducted to examine the impacts of parameter spaces on parameter estimation and testing power. These simulation designs, which were aimed to give a better understanding of model performance under different situations, can provide biologists some empirical evidences to design their experiments.

In the simulation study, the residual variance was calculated under different heritability levels. For the F_2_ design, the genetic variances of various terms can be calculated as follows: 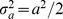, 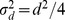, 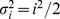, 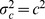, 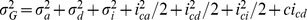, and the broad sense heritability level can be expressed as
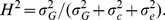
For given genetic parameters and the heritability level, the residual variance can be calculated as 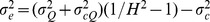, from which the phenotype data can be generated.

The MLEs of the QTL position and effect parameters, based on 200 simulation replicates under different heritability levels and sample sizes, are displayed in Table 2. The square root of mean squared error (RMSE) of parameter estimates are given in parenthesis to show the estimation accuracy. As we expected, the accuracy of parameter estimates increases as the sample size and heritability level increase. For instance, the RMSE of estimated QTL position decreases from 13.28 to 3.81, an 71% increase in accuracy when the sample size increases from 400 to 800 under a fixed heritability level 0.1. The other parameter estimates show the same pattern. If we increase the heritability level when the sample size is fixed, a clear reduction in RMSE can be observed. For example, with 400 samples, the RMSE of estimated QTL position decreases from 13.28 to 4.99, then to 2.77 as 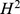 gradually increases from 0.1 to 0.4. From the decreasing RMSEs of the parameter estimates, we observed that simply increasing sample size is less efficient than increasing heritability level in order to increase the precision of parameter estimation. Since high heritability corresponds to small environmental variability [37], reducing environmental variation is of more practically important than just simply increasing sample size.

**Table 2:** The MLEs of the QTL position and effect parameters based on 200 simulation replicates under different heritabilities and sample sizes.

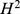	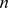	Position 48 cM	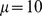	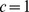	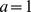	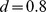	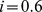	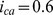	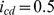	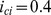	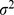
0.1	400	47.93	10.00	0.99	0.96	0.78	-	0.59	0.49	-	3.679
		(13.28)	(0.33)	(0.35)	(0.35)	(0.55)	-	(0.32)	(0.53)	-	(0.22)
	800	47.35	10.01	1.02	0.98	0.77	-	0.60	0.45	-	3.73
		(3.81)	(0.23)	(0.21)	(0.24)	(0.39)	-	(0.22)	(0.33)	-	(0.15)
0.25	400	47.04	10.02	1.00	0.96	0.78	-	0.60	0.50	-	1.99
		(4.99)	(0.17)	(0.18)	(0.16)	(0.26)	-	(0.17)	(0.26)	-	(0.11)
	800	47.65	10.00	1.01	0.98	0.81	-	0.59	0.50	-	2.01
		(2.29)	(0.12)	(0.12)	(0.12)	(0.20)	-	(0.10)	(0.18)	-	(0.08)
0.4	400	48.02	9.99	1.00	0.99	0.81	-	0.60	0.50	-	1.23
		(2.77)	(0.10)	(0.11)	(0.09)	(0.16)	-	(0.10)	(0.17)	-	(0.06)
	800	47.95	9.99	0.99	0.99	0.81	-	0.60	0.51	-	1.24
		(1.78)	(0.07)	(0.07)	(0.08)	(0.12)	-	(0.07)	(0.11)	-	(0.04)

The squared roots of the mean squared errors (RMSE) of the MLEs are given in parentheses.

The locations of the QTL is described by the map distances (in cM) from the first marker of the linkage group. The hypothesized 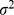 value is 3.81 for 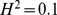, 2.04 for 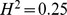 and 1.26 for 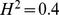.

Note that we did not list the estimation of the imprinting effect 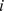 and cyto-imprinting interaction 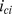, which are not estimable under the F_2_ design. The reason is that the imprinting direction cannot be inferred from the F_2_ design [25]. However, we can still conduct hypothesis test to infer the imprinting effect as well as its interaction with cytoplasmic effect. To further investigate the testing performance of cytoplasmic and imprinting effects, we introduced two proportions, namely 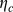 and 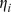, where 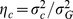 and 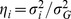. We can evaluate the test power under different cytoplasmic and imprinting effect sizes. Given all other genetic parameter values fixed (as shown in Table 2), simple algebra shows that the cytoplasmic effect 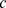 and imprinting effect 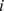 can be calculated for a given value of 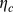 or 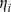, i.e.,
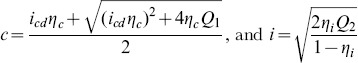
where 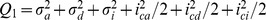, and 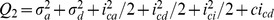.

Based on 1000 simulation runs under different heritabilities, sample sizes and variation proportions, the power of cytoplasmic effect test, imprinting effect test, interaction effects test and additive/dominance effects test are listed in Table 3. As we expect, the test power increases with the increasing of sample size and heritability level. For example, the cytoplasmic testing power increases from 0.663 to 0.905 as sample size increases from 400 to 800, a 36.5% increase in power for fixed 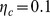 and 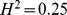. The same pattern is observed for the imprinting test. As the proportion of variance explained by the cytoplasmic and imprinting effect increases, the power increases accordingly. Noted that when both 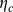 and 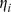 are zeros, the testing power corresponds to the type I error rate for the corresponding factor. From the table we can see that the size of imprinting test is well controlled under different sample sizes and heritability levels. For the cytoplasmic effect, the size is a little inflated under 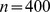, but it gets close to the nominal 5% level as sample size increases to 800, especially under large heritability (e.g., 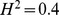). In sum, the simulation evidences show that the model performs reasonably well in both parameter estimation and testing.

**Table 3:** The power of four hypothesis tests based on 1000 samplings under different heritabilities, sample sizes and variation proportions.

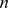	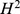	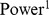			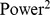			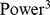	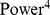
		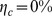	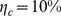	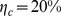	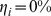	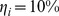	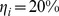		
400	0.10	0.088	0.353	0.528	0.066	0.085	0.089	0.654	0.678
	0.25	0.081	0.663	0.928	0.051	0.263	0.432	0.990	0.992
	0.40	0.064	0.930	0.998	0.050	0.807	0.942	1.000	1.000
800	0.10	0.073	0.464	0.753	0.059	0.100	0.126	0.898	0.927
	0.25	0.066	0.905	0.998	0.047	0.473	0.713	1.000	1.000
	0.40	0.049	0.997	1.000	0.050	0.980	0.997	1.000	1.000

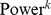
 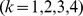 refer to the powers for testing 1) 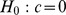 vs 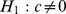; 2) 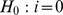 vs 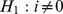; 3) 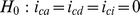 vs 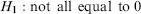; and 4) 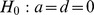 vs 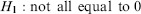, respectively. For a given 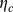, all other effect values are fixed as 0.8 except for 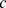, which can be calculated in terms of 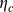 and other parameters. The hypothesized 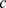 value is 0 for 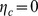, 0.461 for 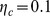 and 0.679 for 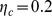. Similarly, the value of 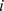, which depends on imprinting effect variation proportion 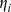, is 0 for 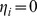, 0.680 for 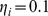 and 1.020 for 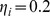.

### A case study

We applied the model to a published F_2_ cross data set based on design 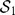 and design 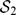 aimed to find QTLs that contribute to variation in quantitative traits related to colitis severity in 1L-10-deficient mice [38]. The data contain 411 F_2_ mice derived from inbred strains, where 203 mice are from design 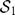 and 208 mice are from design 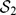. Ninety-one markers were obtained with an average length of ∼15 cM spanning acroos the 19 autosome chromosomes. For more information about the data, the readers are referred to the original paper [38].

**Figure 3:**
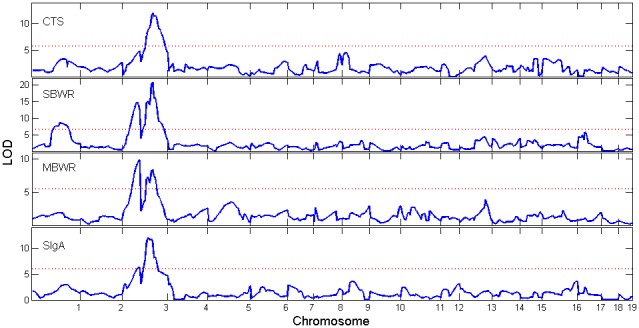
The LOD profiles of the four traits across the 19 chromosomes using the linkage map constructed from microsatellite markers [38]. The genomic positions corresponding to the peak of the curve are the MLEs of the QTL locations.

It has been reported that on average the female chromosome is 25% longer in genetic distance between homologous loci than the male in mice [38]. The sex-specific recombination fraction, expressed as 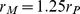, was reconstructed based on the marker information (see Cui et al. for details [25]). The method was applied to four phenotypes, cecum total score (CTS), spleen/body weight ratio (SBWR), mesenteric lymph node(MLN)/body weight ratio(MBWR), and secretory IgA (SIgA) level, where cecum total score was graded by using colitis-related criteria, including severity, hyperplasia, ulceration and the percentage of area involved. Box-Cox transformation was applied to all traits before fitting the Gaussian-mixture model. The genome-wide LOD profile plots for the four phenotypes are shown in Figure 3, where the solid blue curves correspond to the LOD values and the dashed red lines correspond to the 5% genome-wide threshold values out of 1000 permutations. The LOD score is calculated as 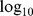
LR, where LR is obtained from equation (9) to test the null hypothesis: 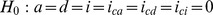.

As shown in Figure 3, one QTL on chromosome 3 is detected for cecum total score trait, two QTLs on chromosomes 3 and one on chromosome 1 are detected for spleen/body weight ratio trait, three QTLs on chromosome 3 are detected for MLN/body weight ratio trait, and two QTLs located on chromosome 3 are detected for SIgA trait. The QTL located at 60.6 cM on chromosome 3 is common to three traits. The one located at 52.6 cM for SIgA trait is very close to it. It is highly possible that it is the same QTL that controls the four traits. Such a pleiotropic effect needs to be further evaluated. It should be mentioned that the QTL detected in the original paper for the four traits is located at 61.8 cM on chromosome 3 [38], which is 1.2 cM away from the one we found. Such a difference may arise because of the capitalization of sex-specific recombination rates and different models fitted.

**Figure 4:**
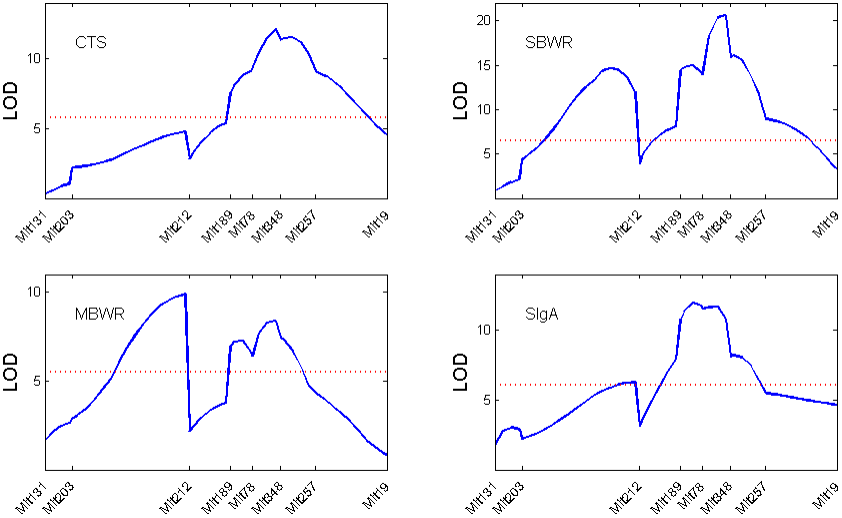
The local LOD profiles of the four traits across chromosomes 3.

In addition to the QTL identified in our analysis and the original paper, some other major QTLs which are not detected in Farmer et al. [14], such as those at 52.6 cM and 38.6 cM on chromosome 3, stand out in our model and therefore need further investigation. Almost all the QTLs on chromosome 3 are clustered together, whose local LOD profiles of four traits are shown in Figure 4.

The marker interval for each QTL is listed in Table 4, which also tabulates the p-values under four different tests for each estimated QTL using permutation tests. From the test results, it can be seen that most QTLs have strong additive and dominance genetic effect, except for the spleen/body weight ratio QTL located at D1Mit156+31.1 cM on chromosome 1. This QTL shows evidence of cytoplasmic, imprinting as well as cyto-nuclear interaction effects (p-values for the three tests are 0.0282, 0.0296 and 0.0073, respectively), but shows no sign of additive and dominance effect. In addition, the MLN/body weight ratio QTL located at D3Mit78+5.6 cM shows evidence of cytoplasmic effect. In summary, we identified one QTL on chromosome 1 with evidence of cyto-nuclear interaction effect and this QTL also shows evidence of cytoplasmic and imprinting effect. Further functional validation is needed to confirm the results.

**Table 4:** List of QTL positions, corresponding marker intervals and p-values under different tests for the four traits.

Trait	Ch	QTL postion	Marker interval	p-value^1^	p-value^2^	p-value^3^	p-value^4^
CTS	3	60.6 cM	D3Mit78-D3Mit348	0.6680	0.3046	0.5005	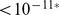
			(D3Mit78+5.6 cM)				
SBWR	3	60.6 cM	D3Mit78-D3Mit348	0.4322	0.6107	0.4502	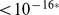
			(D3Mit78+5.6 cM)				
	3	32.6 cM	D3Mit203-D3Mit212	0.2003	0.9502	0.2700	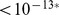
			(D3Mit203+11.4 cM)				
	1	63.9 cM	D3Mit156-D1Mit17	0.0282*	0.0296*	0.0073*	0.8064
			(D1Mit156+31.1 cM)				
MBWR	3	38.6 cM	D3Mit203-D3Mit212	0.3574	0.2651	0.1658	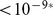
			(D3Mit203+27.4 cM)				
	3	60.6 cM	D3Mit78-D3Mit348	0.0102*	0.9708	0.4831	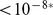
			(D3Mit78+5.6 cM)				
	3	52.6 cM	D3Mit189-D3Mit78	0.2120	0.6421	0.7095	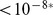
			(D3Mit189+2.9 cM)				
SIgA	3	52.6 cM	D3Mit189-D3Mit78	0.1016	0.6488	0.3195	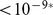
			(D3Mit189+2.9 cM)				
	3	38.6 cM	D3Mit203-D3Mit212	0.7174	0.4051	0.8020	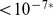
			(D3Mit203+27.4 cM)				

p-value*^k^* 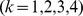 refer to the p-values obtained with the likelihood ratio tests for testing 1) 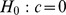 vs 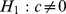; 2) 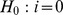 vs 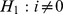; 3) 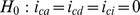 vs 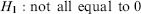; and 4) 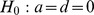 vs 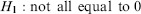, respectively. Significant test results are indicated with the “
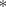
” sign.

## Discussion

The cytoplasmic environment influences the expression of nuclear information in a very complicated way, which is still an unravel mystery to many organisms. For example, Burgess and Husband have demonstrated great maternal contributions to the fitness of mulberry hybrids [39]. While it is an important parent-of-origin effect affecting offspring fitness, genomic imprinting, another source of parent-of-origin effect can also lead to phenotypic variation. Increasing evidence from cytoplasmic substation and cell fusion experiments suggests that weakness of hybrids may connect with the interactions between cytoplasm and nuclear [40], [41], and the evidences about interaction between cytoplasm and imprinting have also been observed [42], [43]. As the source of genetic variation for many traits, these two types of parent-of-origin effects are often confounded, making it difficult to distinguish without proper statistical dissection. Although the role of cross-talk between the two sets of factors on phenotypic variation has been recognized, which genes are involved in the process and in what form they respond to the cytoplasmic changes are still unclear.

In this paper, we developed a statistical model to evaluate the cytoplasmic environment and nuclear gene interaction subject to imprinting effect within the framework of QTL interval mapping. The model that considers eight genetic factors which measure the degree of imprinting, cytoplasmic, additive, dominance effects as well as the interaction effects among them, provides a complete dissection of cyto-nuclear epistasis subject to imprinting effect. A number of hypothesis tests can be performed not only to assess major genetic effect(s) responsible for phenotypic variation, but also to find the statistical evidence for the existence of imprinting, cytoplasmic effect as well as the cyto-nuclear interactions. Simulation study showed relative good performance of the model under the F_2_ design, in which parameters are estimated efficiently with modest heritability and sample size. Low heritability level (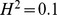) and small sample size (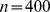) result in large mean squared error of parameter estimation. This result is valuable in practice as we need to be careful about the interpretation of genetic effects obtained in real data analysis when the proportion of variance explained by the QTL is small (i.e., low heritability). Although our model cannot estimate the imprinting effect (so the cyto-imprinting interaction effect) due to the nature of the F_2_ design, existence test of imprinting (or cyto-imprinting interaction effect) can be achieved. Nevertheless, such imprinting estimation problem can be solved under the reciprocal backcross design illustrated in Figure 1.

In the real data analysis, one QTL located on chromosome 1 were found to have significant cytoplasmic, imprinting effect and cyto-nuclear interaction effects for spleen/body weight ratio. Other than that, no imprinting effect was found for all other QTLs, and only one on chromosome 3 that shows cytoplasmic effect for the MLN/body weigh ratio trait. It is worth mentioning that several QTL on chromosome 3 are detected by our model, but they are located relatively close to each other, as shown in Figure 3. For the sake of cautiousness, we reported all of them. However, these detected clustered QTLs may be due to the limitation of interval mapping, which can be overcome by fitting a composite interval mapping model as following,
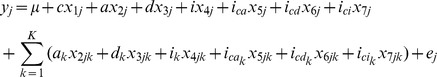
where 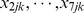 are corresponding variables for the 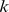
th marker, assuming total 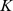 markers are selected for controlling background genetic effect. Although more variables are introduced in the model, theoretically some dimension-reduction techniques such as LASSO, can be applied to implement the variable selection for each marker before fitting them into the final model [44]. The composite interval mapping is know for its improved resolution in QTL detection. Regardless of the potential limitations mentioned above, the integration of cyto-nuclear interactions into the QTL mapping framework provides a testable platform with feasible experimental design for biologists to test the existence of cytoplasmic and imprinting effects, as well as the interactions of interested. The proposed model will have important biological implications with potentials to lift a corner of the great veil of the genetic system.

## Supporting Information

Appendix S1 **Detailed derivation of the EM algorithm.**
(PDF)
