# Correction: Statistical Dissection of Cyto-Nuclear Epistasis Subject to Genomic Imprinting in Line Crosses

**DOI:** 10.1371/journal.pone.0103018

**Published:** 2014-07-11

**Authors:** The PLOS ONE Staff

The order of author affiliations is listed incorrectly. Division of Medical Statistics, School of Public Health, Shanxi Medical University, Taiyuan, Shanxi, China should appear first. The correct list of affiliations is below.

Tao He^2^, Jian Sa^1,2^, Ping-Shou Zhong^2^, Yuehua Cui,^1,2 *^

1 Division of Medical Statistics, School of Public Health, Shanxi Medical University, Taiyuan, Shanxi, China

2 Department of Statistics and Probability, Michigan State University, East Lansing, Michigan, United States of America

* E-mail: cui@stt.msu.edu
