# Supplementary material for: Statistical Dissection of Cyto-Nuclear Epistasis Subject to Genomic Imprinting in Line Crosses
Source: PLoS One. 2014 Mar 18;9(3):e91702. doi: 10.1371/journal.pone.0091702 (PMC3958389; doi:10.1371/journal.pone.0091702)
Supplement: Appendix S1 — Detailed derivation of the EM algorithm. (PDF) [file pone.0091702.s001.pdf]

Appendix S1: EM algorithm

Tao He[‡], Jian Sa[†,‡], Ping-Shou Zhong[‡] and Yuehua Cui[†,‡]

[‡] Department of Statistics and Probability, Michigan State University, East Lansing, Michigan, USA

[†] Division of Medical Statistics, School of Public Health, Shanxi Medical University, Taiyuan, Shanxi, China

Appendix: EM algorithm

The log-likelihood function of Eq.(8) can be expressed as

$$\log L(\mathbf{\Theta}\ |\mathbf{M},\ \mathbf{y}) = \sum_{i=1}^{n_1} \log \sum_{j=1}^{4} \pi_{j|i} f_j(y_i) + \sum_{i=n_1+1}^{n} \log \sum_{j=5}^{8} \pi_{j-4|i} f_j(y_i).$$

By taking derivative with respect to a particular parameter β_k and setting it equal to 0, we can obtain the log-likelihood equation as following,

$$\frac{\partial \log L(\mathbf{\Theta})}{\partial \beta_k} = -\sum_{i=1}^{n_1} \frac{\sum_{j=1}^{4} \pi_{j|i} f_j(y_i) \frac{(y_i - D_j'\beta)}{\sigma^2} D_{jk}}{\sum_{j=1}^{4} \pi_{j|i} f_j(y_i)} - \sum_{i=n_1+1}^{n} \frac{\sum_{j=5}^{8} \pi_{j-4|i} f_j(y_i) \frac{(y_i - D_j'\beta)}{\sigma^2} D_{jk}}{\sum_{j=5}^{8} \pi_{j-4|i} f_j(y_i)} = 0,$$

where $k = 1, 2, ..., 8$, D_j' denotes the jth row of matrix D and D_{jk} is the (j, k)th element of matrix D. Define

$$\begin{aligned} \Pi_{j|i} &= \frac{\pi_{j|i} f_j(y_i)}{\sum_{j=1}^{4} \pi_{j|i} f_j(y_i)}, \quad j = 1, 2, 3, 4 \\ \Pi_{j|i} &= \frac{\pi_{j-4|i} f_j(y_i)}{\sum_{j=5}^{8} \pi_{j-4|i} f_j(y_i)}, \quad j = 5, 6, 7, 8 \end{aligned}$$

as the posterior probability of QTL genotype j for individual i that carries a particular marker genotype. Then the log-likelihood equation is equivalent to

$$\sum_{i=1}^{n_1} \sum_{j=1}^{4} \Pi_{j|i} (y_i - D_j'\beta) D_{jk} + \sum_{i=1}^{n_1} \sum_{j=5}^{8} \Pi_{j|i} (y_i - D_j'\beta) D_{jk} = 0$$

for $k = 1, 2, ..., 8$. Closed form expressions of the estimates of the genotypic values and residual variance in terms of the posterior probabilities can be obtained as follows

$$\hat{\beta}_{k_1} = \frac{\sum_{i=1}^{n_1}\sum_{j=1}^{4}\Pi_{j|i}(y_i - D_{\{j,-k_1\}}\beta_{-k_1})D_{jk_1} + \sum_{i=n_1+1}^{n}\sum_{j=5}^{8}\Pi_{j|i}(y_i - D_{\{j,-k_1\}}\beta_{-k_1})D_{jk_1}}{n},$$

$$\hat{\beta}_{k_2} = \frac{\sum_{i=1}^{n_1}\sum_{j=1}^{4}\Pi_{j|i}(y_i - D_{\{j,-k_2\}}\beta_{-k_2})D_{jk_2} + \sum_{i=n_1+1}^{n}\sum_{j=5}^{8}\Pi_{j|i}(y_i - D_{\{j,-k_2\}}\beta_{-k_2})D_{jk_2}}{\sum_{i=1}^{n_1}(\Pi_{1|i} + \Pi_{4|i}) + \sum_{i=n_1+1}^{n}(\Pi_{5|i} + \Pi_{8|i})},$$

$$\hat{\beta}_{k_3} = \frac{\sum_{i=1}^{n_1}\sum_{j=1}^{4}\Pi_{j|i}(y_i - D_{\{j,-k_3\}}\beta_{-k_3})D_{jk_3} + \sum_{i=n_1+1}^{n}\sum_{j=5}^{8}\Pi_{j|i}(y_i - D_{\{j,-k_3\}}\beta_{-k_3})D_{jk_3}}{\sum_{i=1}^{n_1}(\Pi_{2|i} + \Pi_{3|i}) + \sum_{i=n_1+1}^{n}(\Pi_{6|i} + \Pi_{7|i})},$$

$$\hat{\sigma}^2 = \frac{1}{n}\{\sum_{i=1}^{n_1}\sum_{j=1}^{4}\Pi_{j|i}(y_i - D'_j\beta)^2 + \sum_{i=n_1+1}^{n}\sum_{j=5}^{8}\Pi_{j|i}(y_i - D'_j\beta)^2\},$$

where $k_1 \in \{1, 2\}$, $k_2 \in \{3, 6\}$, $k_3 \in \{4, 5, 7, 8\}$, β_{-k_1} stands for the vector without the k_1th element, and $D_{\{j,-k_1\}}$ stands for the jth row vector without the k_1th element. As mentioned above, the QTL location parameter $\mathbf{\Theta}_l$ can be viewed as a fixed parameter by searching for a putative QTL at every 2cM on a map interval bracketed by two markers throughout the entire linkage map. Therefore, finding the estimated values for QTL genetic parameters $\mathbf{\Theta}_g = (\beta_1, \beta_2, \beta_3, \beta_4, \beta_5, \beta_6, \beta_7, \beta_8, \sigma^2)$=$(\mu, c, a, d, i, i_{ca}, i_{cd}, i_{ci}, \sigma^2)$ is the major objective. At each locus, the EM algorithm is implemented to obtain the MLEs of $\mathbf{\Theta}_g$ as follows:

- Step 1: Give initiate values $\mathbf{\Theta}_g^0 = (\mu, c, a, d, i, i_{ca}, i_{cd}, i_{ci}, \sigma^2)^{(0)}$;
- Step 2: (**E step**) Calculate the posterior probabilities $\{\Pi_{j|i}^{(1)}|\ i = 1, ..., n; j = 1, ..., 8\}$;
- Step 3: (**M step**) Calculate $\mathbf{\Theta}_g^{(1)}$ by using $\Pi_{j|i}^{(1)}$;
- Step 4: Repeat step 2 and step 3 until convergence, and the values at convergence are the MLEs of the parameters.
